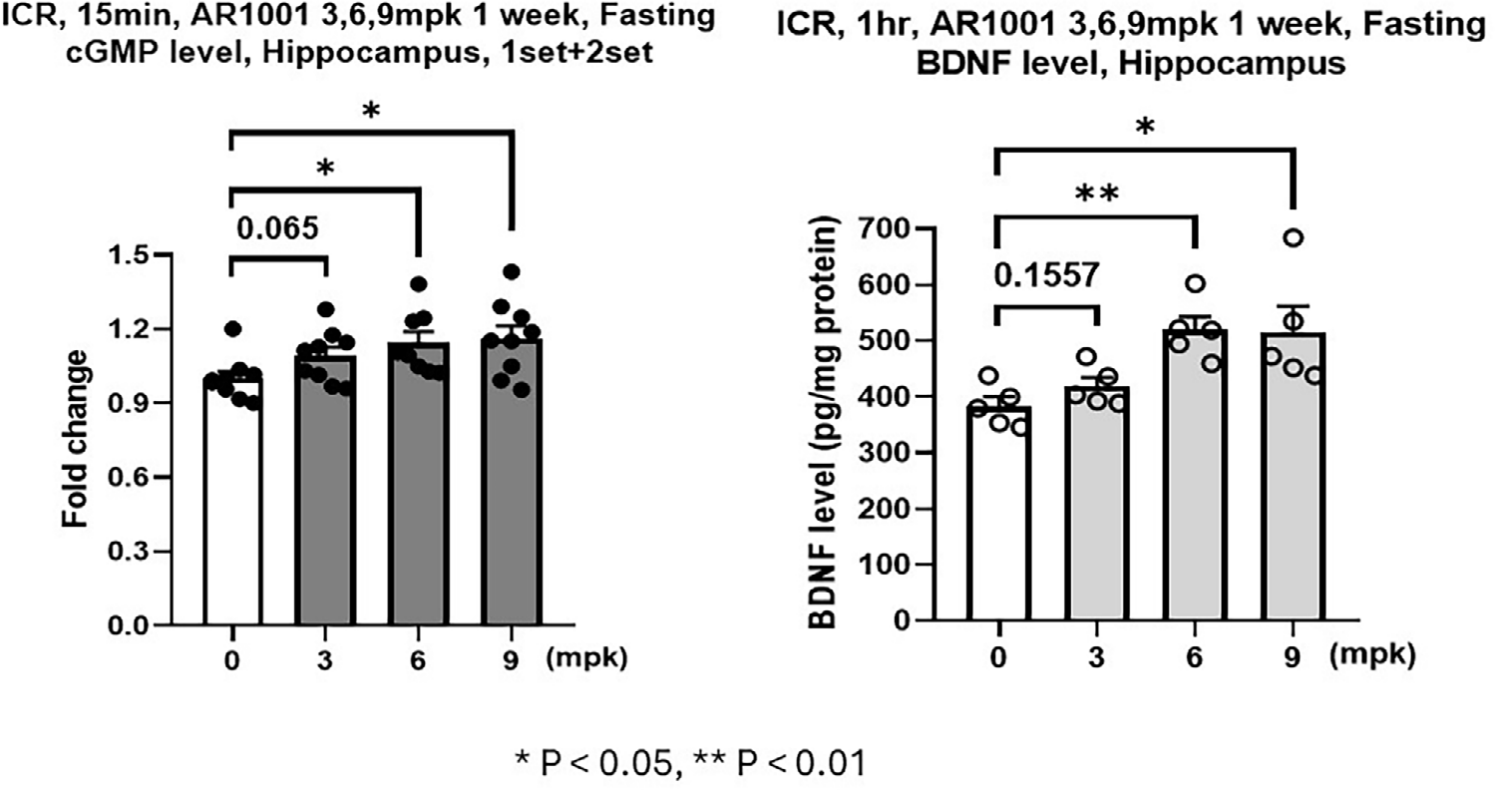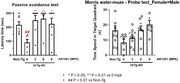# Target Engagement and Therapeutic Effects of AR1001, a Phosphodiesterase‐5 Inhibitor, in Alzheimer’s Disease Models

**DOI:** 10.1002/alz.095314

**Published:** 2025-01-09

**Authors:** Tianyang Xi, Fred Kim, Ju Won Kim, Jun‐Sub Jung, Eunhae Jeon, Hyunji Jo, Ju‐Suk Nam, Dong‐Keun Song, Jai Jun Choung

**Affiliations:** ^1^ AriBio Co., Ltd., San Diego, CA USA; ^2^ AriBio Co., Ltd., Seongnam Korea, Republic of (South)

## Abstract

**Background:**

AR1001 is a specific inhibitor of phosphodiesterase‐5 (PDE5), which degrades cyclic guanosine monophosphate (cGMP). cGMP/cAMP response element‐binding protein (CREB)/brain‐derived neurotrophic factor (BDNF) signaling, which is critical for learning and memory processes, is disturbed in Alzheimer’s disease (AD). AR1001 at the oral dose of 30 mg QD is currently in a global Phase 3 clinical trial in early AD patients (NCT05531526). In this study we sought to determine if AR1001showed target engagement and therapeutic effects in mice at the equivalent dose to that being tested in humans.

**Method:**

An AR1001 oral dose of 6 mg/kg QD in mice is equivalent to an oral dose of 30 mg QD in humans. For the target engagement study, AR1001 was orally administered at 3, 6, and 9 mg/kg QD to overnight‐fasted normal ICR mice for 1 week. Hippocampal levels of cGMP and BDNF 15 min after the last dose were measured by ELISA. To assess the therapeutic effects of AR1001, mice transgenic for familiar AD (3xfAD) were administered 3, 6, and 9 mg/kg QD for 3 weeks, followed by cognitive evaluation using passive avoidance and Morris water maze tests.

**Result:**

In the target engagement study, AR1001 significantly increased hippocampal cGMP and BDNF at doses of 6 and 9 mg/kg QD. In the therapeutic effects study, all 3 doses of AR1001 significantly increased latency in the passive avoidance test compared to untreated controls, and recovered performance to levels of non‐transgenic comparator mice. In the Morris water maze test, AR1001 at 6 and 9 mg/kg significantly increased time spent in the target quadrant compared to untreated controls, and restored performance to levels of non‐transgenic mice.

**Conclusion:**

We demonstrate target engagement of AR1001, a PDE5 inhibitor, by showing an increase in cGMP and BNDF levels in the hippocampus following daily treatment. AR1001 had pronounced effects in recovery of cognitive functions in a transgenic mouse model of AD at a dose equivalent to that being tested in the Phase 3 trial in early AD patients.